# Study of Calcitriol Interaction with the Vitamin D Receptor Using DFT and TD-DFT Calculations

**DOI:** 10.1021/acs.jpcb.5c08130

**Published:** 2026-01-21

**Authors:** Vanessa Regina Miranda, Nelson Henrique Morgon

**Affiliations:** Departamento de Físico-Química, Instituto de Química, 344102 Universidade Estadual de Campinas, Campinas, São Paulo 13083-861, Brazil

## Abstract

Calcitriol, the primary active metabolite of vitamin D, has garnered significant research interest due to its role in several pathologies. However, excessive calcitriol levels or heightened sensitivity of the vitamin D receptor (VDR) can lead to hypercalcemia, motivating the search for analogues that preserve therapeutic activity while reducing adverse effects. Understanding the molecular basis of VDR-calcitriol recognition is therefore essential for rational ligand design. In this study, we applied the ONIOM2­(B3LYP/6–31++G­(2d,p):PM7) hybrid methodology to characterize VDR-calcitriol interactions and identify the most stable conformations while ensuring computational efficiency. Additionally, TD-DFT calculations were performed to explore its electronic properties. We show that calcitriol remains the dominant chromophore and that its main π → π* transition is subtly influenced by interactions with TRP286 and TYR295, providing residue-level insight that is experimentally inaccessible due to the absence of UV–vis data for the holo complex. Furthermore, the calculated binding energy (−11.88 kcal/mol) is consistent with the experimental affinity of the crystallographic VDR construct, supporting the reliability of the predicted binding mode. This integrated analysis of structural, energetic, and electronic features offers new mechanistic insight into VDR-calcitriol recognition and may guide the development of analogues with improved therapeutic profiles.

## Graphical abstract

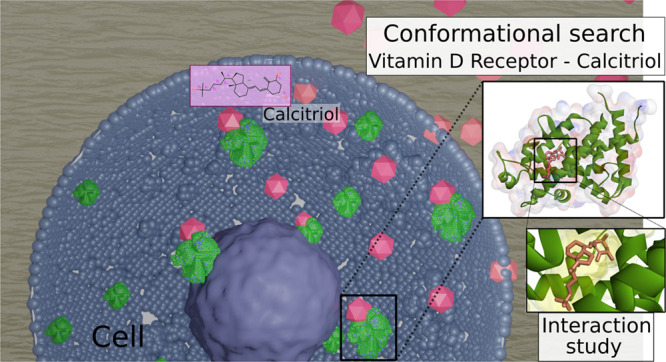

## 1 Introduction

Vitamin D plays a vital role in regulating calcium-phosphate homeostasis within the body,^1,2^ and it is a fat-soluble molecule with hormone-like properties.^3^ Its biological activity manifests following conversion to the active metabolite, lα,25-dihydroxyvitamin D_3_ (calcitriol).^4^ Calcitriol modulates gene expression through activation of the vitamin D nuclear receptor (VDR).^5,6^ Due to its diverse effects, calcitriol has become the subject of extensive research, with investigations exploring its potential in cancer prevention^7^ and treatments,^8^ accelerating the recovery of tuberculosis patients^9^ and protecting the lungs from silica particle-induced injury,^10^ in addition to its metabolic properties and immune function.^2,11^

Despite the diverse beneficial effects of calcitriol, excessive levels or heightened VDR sensitivity to this metabolite can lead to hypercalcemia and Paget’s disease of bone.^12,13^ This has driven the exploration of calcitriol analogues that exhibit adequate VDR interaction profiles, resulting in the therapeutic benefits of calcitriol while minimizing the risk of hypercalcemia. A deeper understanding of the calcitriol interactions within the VDR active site can contribute to the proposal of more effective analogues.^13–18^

Computational studies offer a valuable approach to enhance understanding of ligand-protein interactions. These methods facilitate the determination of the energies associated with the bioactive conformation of the ligand and the surrounding amino acid residues. Additionally, they provide insights into specific interactions, such as hydrogen bonding with individual amino acids within the active site.^19–21^ However, employing high-level quantum mechanics (QM) methods for entire large systems remains impractical due to the significant computational cost and time required.^22,23^

Motivated by the computational limitations of high-level QM methods for large systems, hybrid methods, such as ONIOM (our own n-layered integrated molecular orbital and molecular mechanics) methodology, has emerged as a prominent strategy in recent decades.^24,25^ The ONIOM’s approach considers a partitioning of the system into layers, each calculated with a specific Hamiltonian. This strategy reduces computational costs while allowing results to be obtained with high accuracy.^23,26,27^ Electronic transition studies further advance the understanding of ligand–receptor interactions. Theoretical calculations simulating UV–visible (UV–vis) spectra can provide accurate results when compared to experimental data.^28^ TD-DFT can be employed to computationally obtain such absorption spectra, revealing information about the energy gap between the highest occupied molecular orbital (HOMO) and the lowest unoccupied molecular orbital (LUMO).

Despite the extensive structural characterization of the VDR-calcitriol complex,^14,17,29^ a fundamental gap remains in understanding how the receptor microenvironment modulates the electronic properties of calcitriol. To the best of our knowledge, no experimental UV–vis spectra of the holo complex are available, and no theoretical study has examined the excited-state behavior of calcitriol within the binding pocket or the influence of the protein microenvironment on charge-transfer pathways. In particular, the roles of key residues, such as TRP286 and TYR295, in shaping local excitation, intramolecular charge transfer, and intermolecular charge transfer associated with the calcitriol chromophore remain unexplored. Addressing this gap is essential for establishing a mechanistic link between ligand recognition, interaction energetics, and electronic modulation within the receptor.

This study employed a conformational search using a script developed by us for the ligand-active site complexation. We investigated the specific interactions of the active vitamin D metabolite within the active site of the VDR utilizing Density Functional Theory (DFT) and TD-DFT methodologies. The ONIOM2 methodology, at the B3LYP/6–311++G­(2d,p):PM7 level of theory, was used to describe conformational and energetic aspects concerning the ligand-site interaction, obtaining the most stable energy conformations. Finally, electronic transitions were studied using the UV–vis spectrum and molecular orbital (MO) analysis. The results were validated by comparison with experimental data from the literature. These findings provide important insights for the development of novel calcitriol analogues with improved therapeutic profiles.

## 2 Computational Details

### 2.1 Active Site Definition

The VDR-calcitriol complex was modeled using the crystal structure is from Protein Data Bank (PDB) entry 1DB1,^29,30^ which represents the agonist-bound and biologically active conformation of the VDR ligand-binding domain. This structure is commonly employed in mechanistic and computational studies of VDR activation and provides a reliable template due to its high crystallographic resolution (1.80 Å), which ensures accurate positioning of the residues forming the ligand-binding pocket. Therefore, 1DB1 offers an experimentally well-supported structural framework for the quantum-mechanical analysis performed in this work.

The vitamin D receptor structure comprises a sequence of 259 amino acid residues, and its active site of the VDR, based on the PDB 1DB1, was defined using Molden 7.2 software.^31^ The active site was delineated as all residues within a 6.0 Å radius from the center of mass of the ligand. Residues in this region were protonated at pH 7.4 using the Open Babel program (version 3.0.0).^32^ During this process, amino acid fragments within the site were excised, and hydrogen atoms were added to the terminal groups (i.e., at the −NH and CO- termini) to satisfy valence requirements. Subsequently, these added hydrogens were optimized using the semiempirical PM7 method.

It should be noted that the 1DB1 structure contains no crystallographic water molecules directly occupying the interior of the ligand-binding pocket within the 6.0 Åregion. Instead, four water molecules are located near the outer boundary of this cutoff, positioned along the access channel leading into the cavity. These waters do not directly mediate interactions between calcitriol and the surrounding residues in the pocket. Nevertheless, dynamic solvation effects may modulate the persistence of these contacts in a biological environment, and such effects lie beyond the scope of the present QM-based study.

### 2.2 Conformational Analysis

The conformational analysis of the ligand within the active site was performed using an adapted computational code previously developed by our research group and successfully applied in prior studies.^33,34^ This code facilitates the insertion of calcitriol into the VDR active site, accounting for translational movements of the ligand.

The conformational search of the active form of vitamin D inside the active site began with a relaxed potential energy surface scan, varying dihedral angles in 90° increments over a 0° to 360° range. Simultaneously, the ligand was subjected to three-dimensional translational movements along the *x*, *y*, and *z* axes. At each variation of eight selected calcitriol dihedral angles (Figure 1), systematic sampling was performed within the active site while keeping the protein atoms fixed.

**Figure 1:**
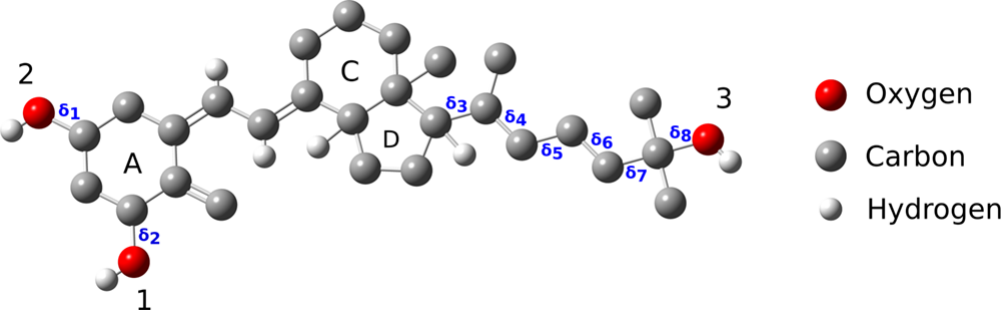
Eight dihedral angles of calcitriol selected for conformational scanning.

This procedure generated a diverse set of ligand conformations within the active site. Subsequently, each calcitriol conformation, along with the hydrogen atoms from the active site, was optimized using the semiempirical PM7 method, while keeping the heavy atoms of the VDR fixed. This optimization aimed to identify the most stable structures based on statistical thermodynamics analysis. Following the geometry optimizations, vibrational frequency calculations were performed to confirm that the structures correspond to minima on the potential energy surface, as indicated by the absence of imaginary frequencies. The population analysis of the conformations was conducted using the Boltzmann distribution (eq 1), which accounts for the individual energy contribution (percentage) of each conformation.

$$P_{i}=\frac{e^{-\Delta G_{i}/RT}}{\sum_{j=1}^{N}e^{-\Delta G_{j}/RT}}$$ 1

### 2.3 ONIOM

ONIOM is a hybrid multilayer extrapolation method developed by Morokuma and collaborators,^24,25^ designed to describe large molecular systems, such as biomolecules, by partitioning them into two or more layers (levels). Each layer is computed using an individualized Hamiltonian, and the results are combined through an extrapolation scheme that accounts for the entire system, yielding a more accurate total energy. The most chemically important region is treated at a higher level of theory, while the remaining parts are described at a lower level.

In this study, we employed the ONIOM2­(QM/QM) method as a two-layer model to determine the optimal conformation of calcitriol within the VDR residue pocket and to investigate the system energy of the VDR active site complexed with calcitriol. To balance accuracy and computational cost, the ONIOM2­(QM/QM) layers were defined using B3LYP/6–31++G­(2d,p)­for the high-level region (calcitriol), whereas the VDR active site was treated at the lower level using the semiempirical PM7 method, with the heavy atoms of the active site kept fixed.

This combination has been widely and successfully applied in quantum chemical studies of protein–ligand systems, including SARS-CoV-2 spike-ACE2 complexes, GH116 β-glucosidase inhibitors, and acetylcholinesterase-2-PAM assemblies.^35–37^ The system was treated with Grimme’s D3 empirical dispersion correction via the IOp­(3/124 = 50) keyword in Gaussian 16, Revision A.03.^38^ Each optimized geometry was verified as an energy minimum by frequency analysis. This procedure provides an approximate high-level energy value for the full system, corresponding to the B3LYP/6–31++G­(2d,p) level, as expressed in eq 2.

The energy function for the ONIOM2 method is defined as the sum of the high-level energy of the model system (*E*_high,model_) and the low-level energy of the real system (*E*_low,real_), minus the low-level energy of the model system (*E*_low,model_), as expressed by the following equation:^23,26^

$$E_{\mathrm{high},\mathrm{real}}\approx E_{\mathrm{ONIOM2}}=E_{\mathrm{high},\mathrm{model}}+E_{\mathrm{low},\mathrm{real}}-E_{\mathrm{low},\mathrm{model}}$$ 2

Subsequently, population analysis was performed on the extrapolated energies obtained via the ONIOM2 method for each system (calcitriol and VDR). This analysis allowed the identification of the most stable conformations, characterized by the highest percentage contribution in the population distribution.

### 2.4 Noncovalent Interaction

Noncovalent interactions (NCI)^39,40^ index is a technique that utilizes the electron density (ρ) to generate a gradient of isosurface, representing intermolecular and intramolecular interactions. This feature makes NCI a valuable tool for investigating as to the real space of noncovalent interactions within chemical and biological systems.

To investigate the interactions between calcitriol and the VDR active site residues, NCI analysis was employed through the NCIPLOT program, version 4.2.1 alpha.^39–41^ The NCI calculations were performed on the ligand and active site structure obtained previously through the ONIOM2 method. The NCI analysis yielded an isosurface map of the interactions between the ligand and the amino acid residues within the active site. The resulting isovalue isosurface were rendered using VMD software, version 1.9.4a55.^42^ The two-dimensional plots of the Reduced Density Gradient (RDG or s) as a function of sign­(λ_2_)­ρ were generated using Gnuplot, version 5.4.^43^

### 2.5 Binding Energy Evaluation

The global binding energy between calcitriol and the VDR active site was estimated using a Dreiding force-field-based energy decomposition.^44^ The geometries of the complex, the isolated binding site, and the isolated ligand were optimized, and their energies were computed independently. The binding energy was then evaluated as

$$\mathrm{Binding}\;\mathrm{Energy}=E_{\mathrm{Complex}}-(E_{\mathrm{Active}\;\mathrm{site}}+E_{\mathrm{Ligand}})$$ 3

This procedure provides a classical, force-field-based estimate of the overall stabilization of the ligand within the receptor, complementing the quantum mechanical residue-level interaction analyses.

### 2.6 TD-DFT

Excited-state calculations were carried out for the most energy-stable system obtained from the ONIOM2 ground-state optimization. All TD-DFT computations were performed at the single-layer, without employing any ONIOM excited-state formalism or electrostatic embedding. To improve the description of the complexes, TD-DFT studies were employed including exact long- range exchange, as implemented in the ωB97X functional. This functional was used with the basis set 6–311++G­(2df,p), and 45 lowest singlet excited states were calculated. Molecular orbital energies and UV–vis spectra were studied using the ground-state geometry. To gain further insight into the electronic transitions in calcitriol, the molecule was extracted from the ONIOM-optimized complex and subjected to separate TD-DFT calculations. This step allowed for the independent analysis of the HOMO and LUMO orbitals of calcitriol.

Subsequently, the complex formed by the active form of calcitriol and specific amino acid residues from the VDR active site, identified as being in close proximity to the key HOMO and LUMO regions of the calcitriol molecule, was subjected to TD-DFT calculations at the same level of theory (ωB97*X*/6–311++G­(2df,p), with 45 excited states). This comparative analysis aimed to determine potential perturbations in the UV–vis spectrum and electronic transitions arising from the formation of the complex with these residues.

Given the absence of experimental UV–Vis data for the holo VDR-calcitriol complex, the validation of the computational strategy employed in this study was previously es- tablished for the calcitriol structure (CAS Number: 32222-06-3).^45^ It was first optimized with frequencies calculated using the level of theory DFT-B3LYP/6–31G­(d) and then followed by the DFT-B3LYP/6–3++G­(2d,p), both in ethanol solvent (ε = 24.852), using the Solvation Model Based on Solute Electron Density (SMD) model^46^ and explicit solvent. Electronic transitions were calculated at the TD-ωB97*X*/6–311++G­(2df,p) level of theory. This system has available experimental UV–visible spectral data,^47^ enabling direct comparison and validation of the computational results.

The total electron density surfaces of calcitriol and the aromatic amino acid residues were analyzed along with their electrostatic potential values at the ground-state geometries using GaussView version 5.0.^48^ The UV–visible electronic spectra were plotted using Molden 7.2 software.^31^ Within this software, half-height adjustments were applied to the bands, and the spectra were subsequently normalized. All calculations were performed using the Gaussian16 program, Revisions A.03 and C.01.^38,49^ Computational simulations in this study were carried out on the Coaraci Supercomputer at the Center for Computing in Engineering and Sciences, University of Campinas (Unicamp).

## 3 Results and Discussion

DFT and TD-DFT were employed to investigate the geometrical and electronic properties of calcitriol complexed with amino acid residues within the active site of the vitamin D receptor.

### 3.1 Active Site Definition and Conformational Analysis

The crystal structure of the VDR was obtained from the Protein Data Bank (PDB: 1DB1)^29,30^ (Figure 2A). The active site was defined by selecting all residues within a 6.0 Å radius from the center of mass of calcitriol, following the approach described in the literature^33,50^ (Figure 2B). This selection yielded an active site comprising a total of 788 atoms.

**Figure 2:**
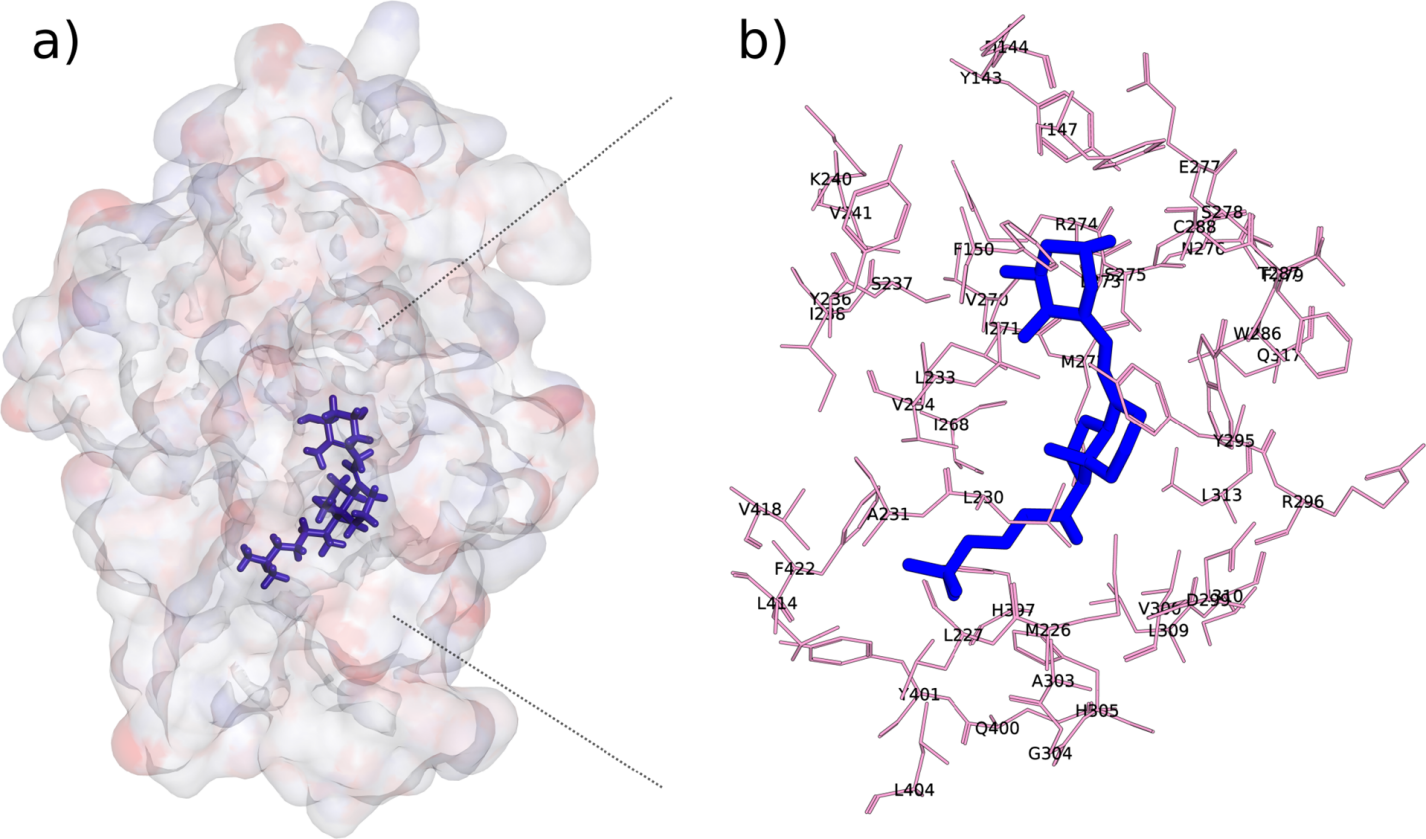
(a) Crystal structure of the vitamin D receptor (PDB: 1DB1) in complex with calcitriol (blue); (b) detailed view of calcitriol (blue) surrounded by key amino acid residues (purple) within 6.0 Å in the binding site.

The ligand structure was subjected to a relaxed potential energy surface (PES) scan of eight dihedral angles within the active site. Conformational analysis identified 26 distinct calcitriol conformations within the VDR active site. These structures were optimized using the semiempirical PM7 method, with relaxation permitted only for ligand and hydrogen atoms while keeping heavy atoms of the active site constrained. Postoptimization energy calculations and statistical analysis of the energy distribution revealed 12 conformations with the highest individual energy contributions. Conformation 25 exhibited the largest energy contribution (8.9%), distinguishing it significantly from the other conformers (Table 1). With the exception of conformation 9, which showed the lowest contribution, energy distributions across most conformations were remarkably similar.

**Table 1:** Boltzmann-Weighted Energy Decomposition Analysis for Calcitriol Conformations in the VDR Binding Site

system	25	17	20	7	18	1	2	5	6	11	16	9
contribution (%)	8.9	8.5	8.4	8.3	8.2	8.1	8.1	8.0	8.0	7.9	7.9	2.9

### 3.2 ONIOM Calculations

The 12 conformations were analyzed using the ONIOM2­(B3LYP/6–31++G­(2d,p):PM7) methodology to determine extrapolated electronic energies. In this approach, both the ligand and active site received quantum mechanical treatment, with calcitriol being computed at a significantly higher level of theory.

The calculations identified three thermodynamically stable conformations: 07 (Figure 3B), 09 (Figure 3C), and 25 (Figure 3D). These conformations exhibited superior complexation energy stability between calcitriol and the VDR active site residues compared to other conformers.

**Figure 3:**
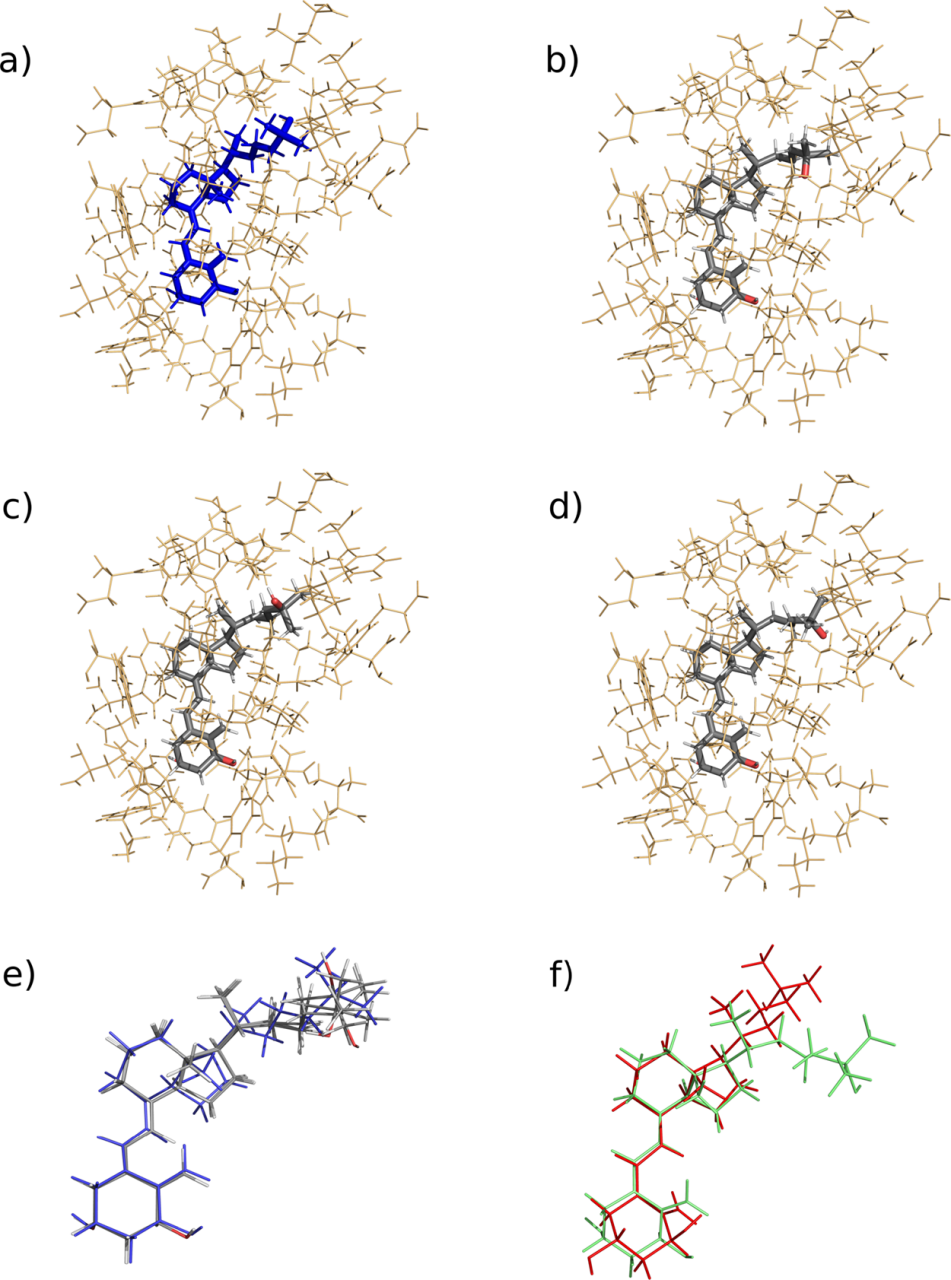
Structural comparison of calcitriol conformations in the VDR binding site. (a) Experimental structure from PDB: 1DB1 (residues within 6.0 Å of calcitriol). (b–d) Three most stable ONIOM2 - optimized conformations (B3LYP/6–3++G­(2d,p):PM7): (b) conformation 07, (c) conformation 09, and (d) conformation 25. (e) Structural superposition of theoretical conformations (gray) aligned with the experimental structure (blue). (f) Conformational comparison between VDR-bound calcitriol (red, conformation 25) and its gas-phase optimized structure (green, at B3LYP/6–31++G­(2d,p) level).

The calcitriol conformations were superimposed onto the experimentally determined structure (PDB: 1DB1),^29,30^ as shown in Figure 3E. This structural alignment reveals close conformational similarity to the crystal structure, with minor deviations localized primarily in the flexible side chain region of calcitriol.

Single-point energy and frequency calculations were performed at the B3LYP/6–31++G­(2d,p) level for both the isolated calcitriol conformations (extracted from the VDR active site and the experimental structure). Table 2 presents the energy differences (Δ*E*) between the calcitriol conformations obtained from QM/QM calculations and the experimental structure from PDB: 1DB1. Among these, conformation 25 exhibited the smallest energy deviation relative to the experimental structure.

**Table 2:** Structural and Energetic Comparison of Calcitriol Conformations

geometry	Δ*E* ^a^	RMSD^b^
experimental^c^	0	0
07	49.34	6.325
09	49.30	6.371
25	46.46	6.312

a ΔE in kcal/mol.

b Root mean square deviation in Å.

c Experimental geometry (PDB: 1DB1).

The root-mean-square deviation (RMSD) analysis revealed close values for all calcitriol conformations, with conformation 25 exhibiting the smallest deviation (Table 2). This suggests that conformation 25 represents the optimal accommodation of active vitamin D within the VDR binding cavity, demonstrating similar interactions with active site residues compared to the experimental structure. Consequently, we selected conformation 25 for subsequent interaction and TD-DFT calculations.

Figure 3F illustrates the result of the influence of intermolecular interactions on calcitriol conformation within the VDR active site. The overlay compares the bioactive conformation (red, structure 25) with the gas-phase optimized structure (green, DFT-B3LYP/6–31++G­(2d,p)), revealing significant conformational differences. These findings align with previous studies of calcitriol analogues using Fragment Molecular Orbital calculations^51^ and other ligand–receptor systems.^52,53^

The VDR-bound conformation of calcitriol (structure 25) adopts a B-chair configuration, with the 1-hydroxyl group equatorial and 2-hydroxyl group axial. This conformation matches the crystallographic observations by Rochel et al.^29^ and solution-phase studies by Bouillon et al.,^54^ who reported an equilibrium between A- and B-chair conformations.

The conjugated polyene system between rings A and C fits precisely within the hydrophobic channel, providing strong ligand anchoring. In contrast, the flexible side chain, surrounded by nonpolar residues, exhibits significant conformational variability due to its single-bond architecture (Figure 3E). This flexibility, combined with the ligand’s size, accounts for the slightly higher RMSD values observed in our conformational search.

The curved conformation observed in our study results from two key factors: (1) the constrained accommodation of the conjugated triene system within the hydrophobic channel, which enforces nonplanar geometry as previously reported,^29^ and (2) specific interactions between calcitriol’s side chain and surrounding residues. This curvature becomes particularly evident when comparing the bioactive conformation with the gas-phase optimized geometry (Figure 3F), where the absence of protein constraints yields a significantly more planar structure.

### 3.3 Noncovalent Interaction Analysis

The Non-Covalent Interaction (NCI) analysis was performed to characterize the intermolecular forces stabilizing the VDR-calcitriol complex, using the most stable conformation (system 25) identified in our conformational search.

The NCI isosurfaces revealed the spatial distribution of interactions between calcitriol and VDR binding site residues in real space. The color-coded scheme represents:

- Blue: Strong attractive interactions (sign­(λ_2_)­ρ < 0), such as hydrogen bonds;
- Green: van der Waals interactions (sign­(λ_2_)­ρ ≈ 0);
- Red: Repulsive/steric interactions (sign­(λ_2_)­ρ > 0).

The Reduced Density Gradient (RDG) versus sign­(λ_2_)­ρ plot (Figure 4A) reveals two prominent interaction regimes. In the lower region of the graph, sharp and intense blue spikes are observed, which correspond to hydrogen bonding interactions. Additionally, a broad and diffuse green region dominates the central portion of the plot, indicating the prevalence of van der Waals interactions across the system.

**Figure 4:**
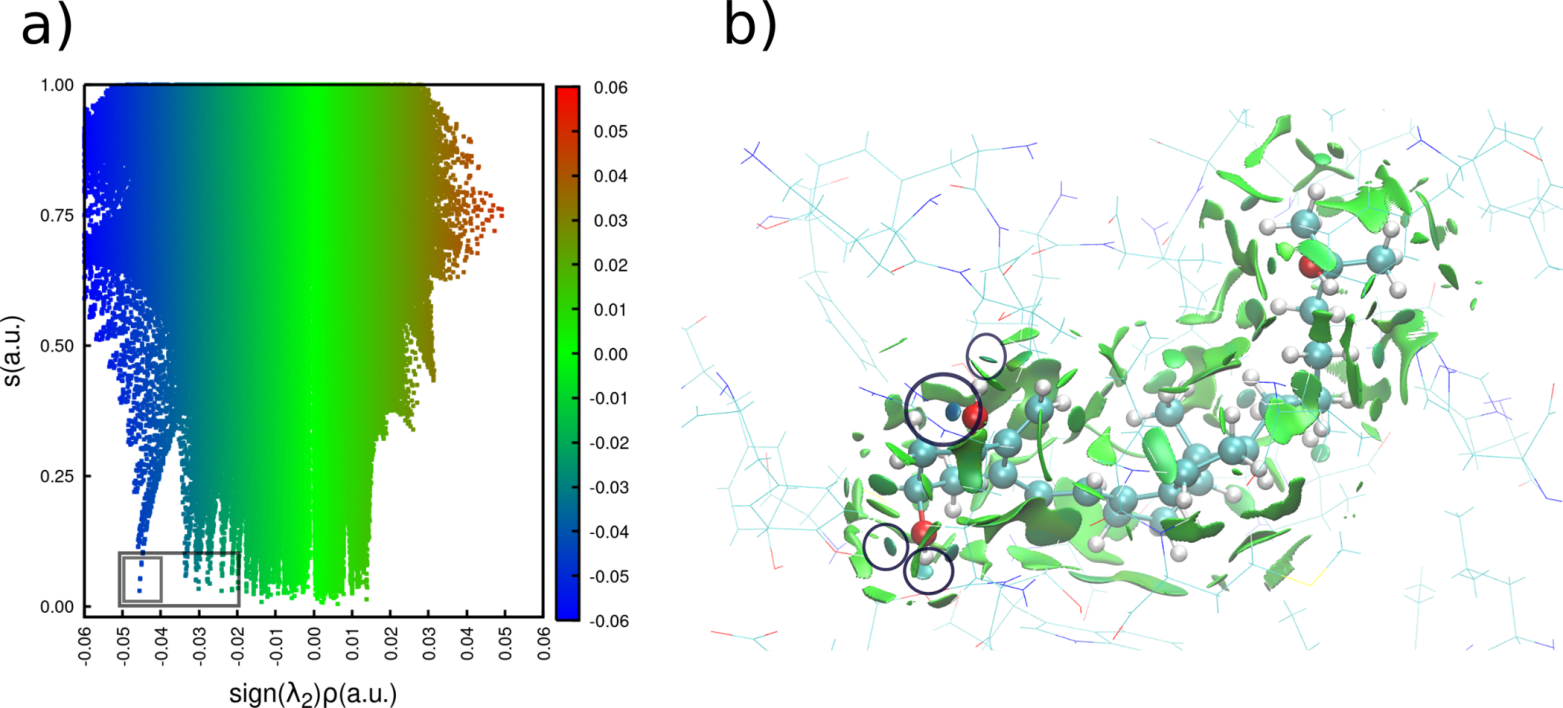
Noncovalent interaction (NCI) analysis between the lowest-energy conformation of calcitriol and binding site residues of the vitamin D receptor (within 6.0 Å). (a) Scatter plot of reduced density gradient (RDG; *s*) versus sign­(λ_2_) ρ, showing electron density regions from −0.06 to 0.06 au (b) NCI isosurfaces (RDG = 0.3 au): Hydrogen bonds (blue) between calcitriol hydroxyl groups and residues, and hydrophobic interactions (green) between residues and calcitriol.

The hydrogen bonding interactions are prominently displayed in the lower left region of the RDG plot (Figure 4A), with a highlighted box indicating the most attractive interactions and a secondary box emphasizing the strongest hydrogen bond. These interactions correspond to the intense blue isosurfaces shown in Figure 4B, with spatial distributions that match the circled regions in the 3D interaction map.

These specific interactions (Figure 4B) demonstrate how VDR achieves ligand recognition through precise positioning of polar residues that complement calcitriol’s hydroxyl group arrangement.

#### 3.3.1 Hydrogen Bonding and van der Waals Interactions

The NCI analyses showed in the Table 3 summarizes the key hydrogen bonding interactions, including donor and acceptor atom identification, participating amino acid residues, and interaction strengths. Our analysis reveals crucial hydrogen bonds between:

- The 1-hydroxyl group of calcitriol’s A-ring and the ARG274 residue of VDR;
- The 1-hydroxyl group of calcitriol’s A-ring and the SER237 residue of VDR;
- The 2-hydroxyl group of calcitriol’s A-ring and the TYR143 residue of VDR;
- The 2-hydroxyl group of calcitriol’s A-ring and the SER278 residue of VDR.

**Table 3:** Hydrogen Bonding Interactions between Calcitriol and Binding Site Residues of the Vitamin D Receptor Identified via NCI Analysis

ligand (atom)	residue (atom)	role (donor → acceptor)
H (−OH 1, ring A)	O (hydroxyl, SER237)	ligand → protein
O (−OH 1, ring A)	H (guanidinium, ARG274)	protein → ligand
O (−OH 2, ring A)	H (phenol, TYR143)	protein → ligand
H (−OH 2, ring A)	O (hydroxyl, SER278)	ligand → Protein

The NCI analysis revealed that the hydrogen bond between calcitriol’s 1-hydroxyl group and ARG274 presents the most favorable interaction profile, as indicated by the intense blue isosurface (Figure 4). Additional crucial hydrogen bonds were observed between the ligand’s hydroxyl groups and residues SER237, TYR143, and SER278. These polar interactions collectively ensure optimal ligand accommodation within the VDR binding pocket, enhance complex stability, and mediate specific molecular recognition. These computational findings corroborate crystallographic data from the VDR-calcitriol complex (PDB: 1DB1)^29^ and confirm the fundamental role of hydrogen bonding in ligand-protein recognition.^55^

The NCI results further revealed extensive van der Waals interactions across the calcitriol-VDR interface (Figure 4B), mediated primarily by hydrophobic residues in the binding cavity.^29,56^ Particularly notable is the pronounced hydrophobic channel that accommodates the conjugated triene system (between rings A and C), featuring strong interactions with aromatic residues TRP286 and TYR295, along with complementary contacts to LEU233 and SER275. These dispersive interactions serve to anchor the triene moiety deeply within the binding pocket while providing substantial stabilization energy to the complex.

The combined analysis demonstrates how hydrogen bonds and van der Waals forces work synergistically to drive conformational selection and induced-fit binding of calcitriol’s bioactive conformation. This dual stabilization mechanism explains the high binding affinity observed experimentally and provides molecular-level insights into VDR’s ligand recognition specificity, consistent with both our computational results and crystallographic evidence.^29^ The complementary nature of these interactions–with polar groups mediating precise positioning and hydrophobic contacts providing substantial binding energy–represents a classic example of biological molecular recognition.

In addition to the residue-resolved analysis, we estimated the overall binding energy between calcitriol and the VDR binding site using a Dreiding force-field.^44^The resulting binding energy, −11.88 kcal/mol, is in remarkable agreement with the experimental binding free energy inferred from the reported dissociation constant of the VDR-calcitriol complex (*K*_d_ = 0.37 ± 0.05 nM;^29^ Δ*G*_bind_ ≈ −12 kcal/mol). This quantitative consistency supports the calculated binding mode and reinforces the role of the identified hydrogen-bond network and hydrophobic channel in stabilizing the ligand within the receptor.

Although the computed interaction energies are consistent with the experimental *K*_d_ reported for the VDR-calcitriol complex, full thermodynamic validation would require calorimetric or ITC measurements to directly determine Δ*H*, Δ*S*, and Δ*G*. Such experimental characterization lies beyond the scope of the present theoretical study.

### 3.4 TD-DFT Calculations

The methodology for electronic transition studies was validated by computing the UV–vis spectrum of calcitriol in explicit ethanol solvent using the SMD continuum model at the TD-ωB97*X*/6–311++G­(2df,p) level of theory. The calculated maximum absorption wavelength (λ_max_) of 268.1 nm showed excellent agreement with the experimental value of 265 nm in ethanol,^47^ confirming the reliability of our computational approach. To the best of our knowledge, experimental UV–vis spectra for the holo VDR-calcitriol complex have not been reported in the literature. Therefore, our analysis of electronic transitions focuses on a description of how the protein environment, particularly the aromatic residues TRP286 and TYR295 located near the polyene chromophore, perturbs the excited-state properties of calcitriol. This approach builds upon the experimentally validated UV–vis spectrum of free calcitriol in solution, while extending it through TD-ωB97X calculations that capture residue-specific effects not accessible experimentally.

Analysis of calcitriol’s electronic transitions identified aromatic residues TRP286 and TYR295 as prime candidates for detailed study due to their spatial proximity to the ligand’s chromophoric region and known sensitivity to electronic environment changes. Figure 5 presents the four frontier molecular orbitals with their corresponding energies, including the HOMO–LUMO gap (Δ*E*_HL_) for each structure. Further examination of the calcitriol-TRP286-TYR295 complex in Figure 6 reveals ten molecular orbitals involved in these interactions.

**Figure 5:**
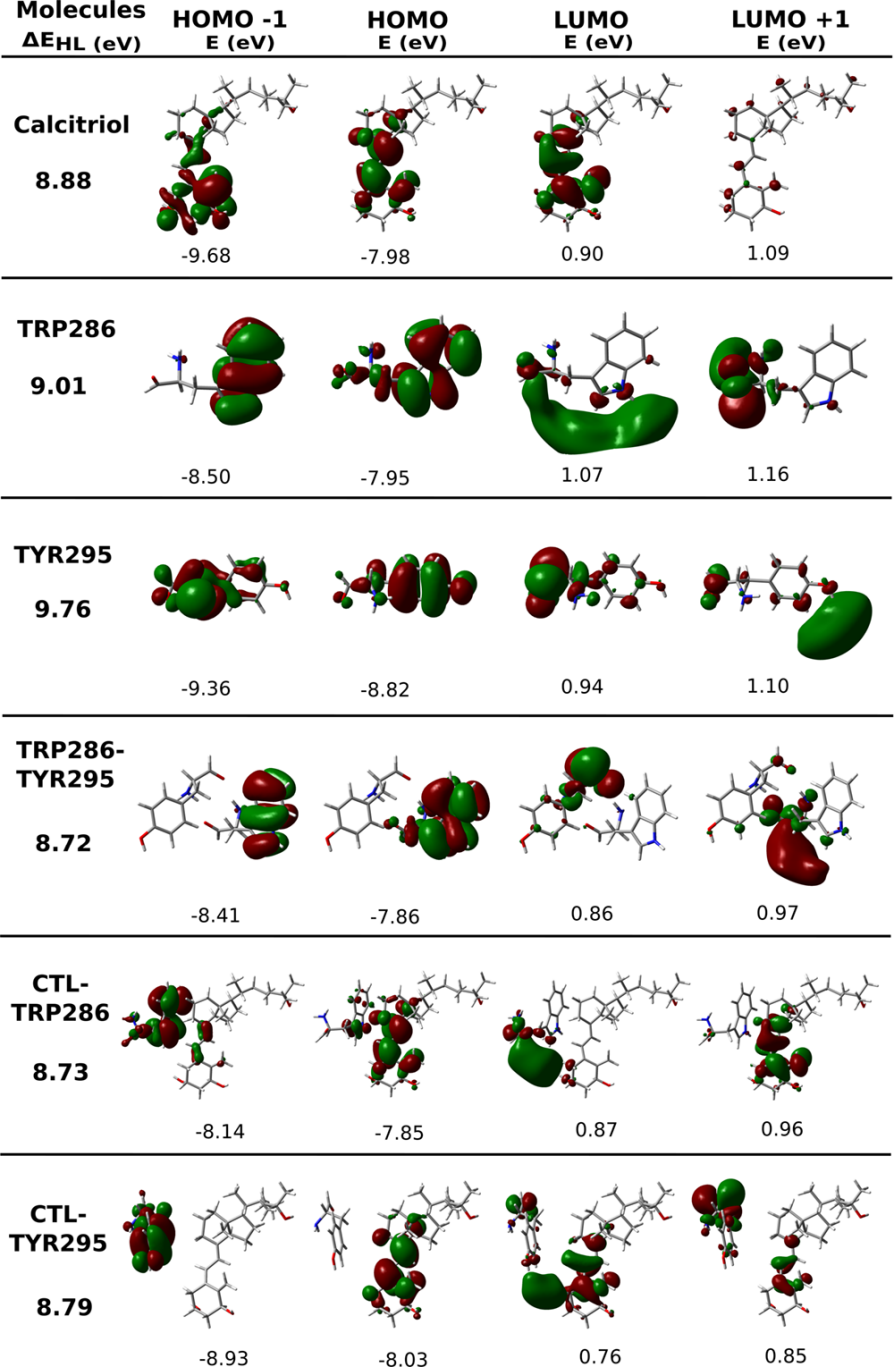
Ground-state molecular orbitals (energies labeled) and HOMO–LUMO gaps for isolated calcitriol and key aromatic residues from the VDR binding site, computed at the ωB97*X*/6–311++G­(2df,p) level of theory.

**Figure 6:**
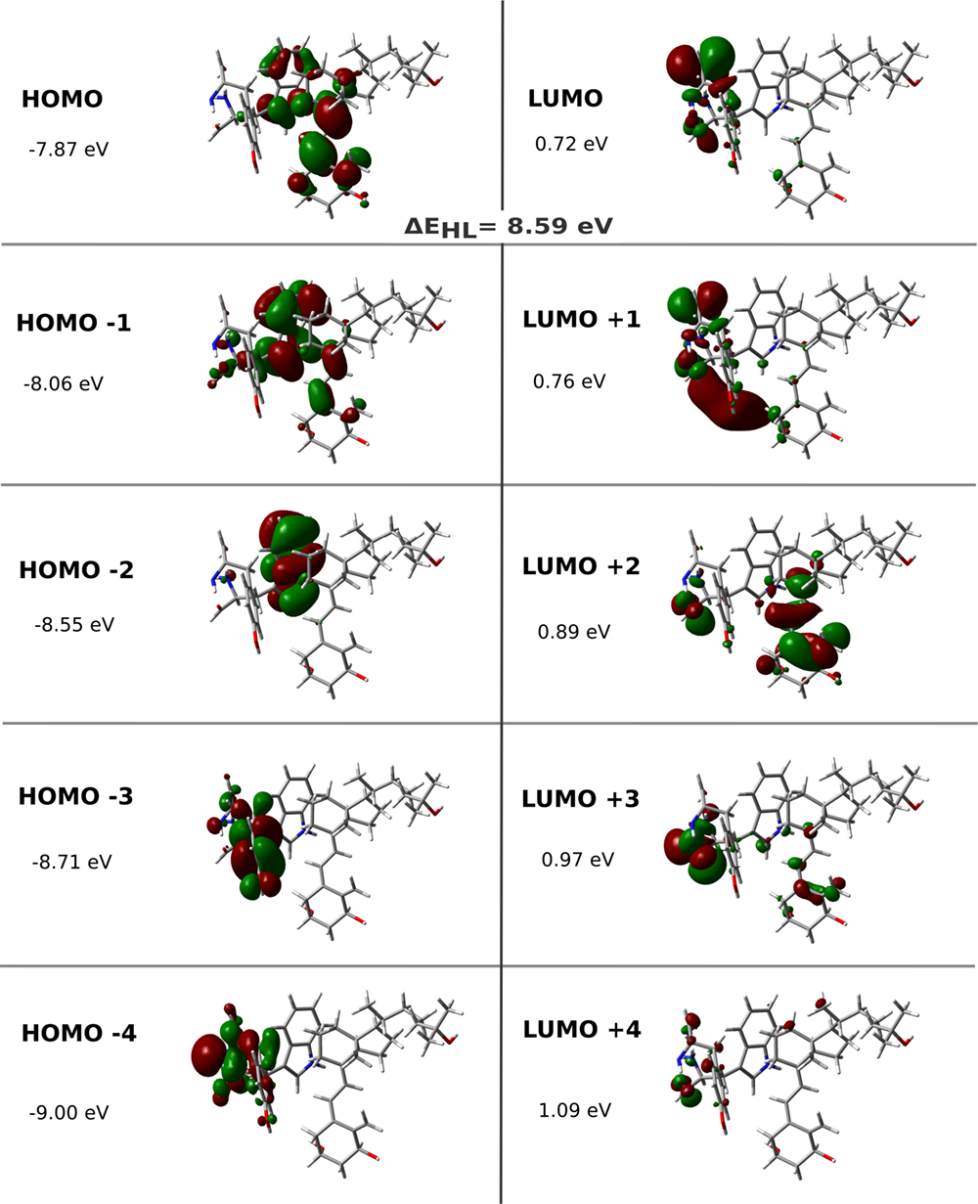
Molecular orbitals and energies of electronically excited states for the complex of calcitriol with VDR binding site residues TRP286 and TYR295, including HOMO–LUMO gaps, calculated at the TD-ωB97*X*/6–31++G­(2df,p) level of theory.

Notably, the Δ*E*_HL_ values for the isolated aromatic residues are higher than when both residues are present together (TRP286–TYR295). The isolated TYR295 exhibits a higher Δ*E*_HL_ (9.76 eV) compared to the combined TRP286–TYR295 system (8.72 eV). The energy gap in this dimeric residue system resembles values observed for individual residue complexes with calcitriol. Most significantly, when calcitriol complexes with both aromatic residues near its electronic transition region, we observe a reduced Δ*E*_HL_ relative to either isolated calcitriol or single-residue complexes. This modest but consistent decrease in the HOMO–LUMO gap suggests enhanced probability of intermolecular charge transfer between the aromatic residues and calcitriol, potentially contributing to the stabilization of the ligand–receptor complex.

Analysis of the spatial electron density distribution associated with the HOMO–LUMO transitions in calcitriol (Figure 5) reveals predominantly local excitation (LE) character, concentrated on the conjugated triene system. When calcitriol complexes with TRP286, the HOMO exhibits enhanced electron density over the triene moiety with slight delocalization extending to TRP286’s indole ring. Conversely, the LUMO is primarily localized on the amino acid, particularly beyond its side chain, indicating strong intermolecular charge transfer alongside weaker intramolecular charge transfer (ICT) within calcitriol and TRP286.

In the calcitriol-TYR295 complex, the HOMO remains fully localized on the ligand’s triene system, while the LUMO shows intense but diffuse density primarily within the same region (characteristic of LE), with minor extension to TYR295. This distribution suggests dominant ICT with minor intermolecular charge transfer (ECT) contribution.

The LUMO in the calcitriol-TRP286 complex exhibits diffuse, delocalized charge originating from TRP286, while in calcitriol-TYR295 the charge delocalization direction reverses (Figure 5). Both manifest as concentrated electron density between interacting molecules. This feature disappears when calcitriol complexes with both aromatic residues (Figure 6). Here, the HOMO localizes over calcitriol and TRP286’s indole ring, while TYR295 shows negligible density contribution. The LUMO predominantly resides on TYR295, indicating pronounced intermolecular charge transfer character in the HOMO–LUMO transition. This configuration reveals strong spatial separation between donor (calcitriol-TRP286) and acceptor (TYR295) orbitals, with ionization potential primarily associated with calcitriol-TRP286 and electron affinity with TYR295. Consequently, minimal orbital overlap exists between HOMO and LUMO.

Table 4 summarizes electronic transition properties for individual molecules and complexes, listing the most intense S_0_ → S_*n*_ (*n* = 1–45) transitions. Isolated calcitriol exhibits the highest oscillator strength (*f*), which decreases by ∼17% upon complexation with TRP286 and TYR295. The transition dipole moment (μ_tr_) of free calcitriol similarly reduces by over 50% in the complex. The predominant transition in the ternary complex is H → L + 2 (Table 4), displaying strong LE character within calcitriol’s polyene region combined with charge transfer (CT) to the central region of the TRP286 residue (Figure 6), contributing 61% to the transition.

**Table 4:** Electronic Transition Properties of Calcitriol and Binding Site Aromatic Amino Acids (TRP286 and TYR295) Calculated at the TD-ωB97*X*/6-311++G­(2df,p) Level of Theory

molecules	state	λ_max_, nm	*f. osc.*	μtr, D	predominant transitions	character (HOMO–LUMO)^a^	%^b^
calcitriol	S_1_	266.9	0.5465	4.7610	H → L^c^	ICT	98
TRP286	S_9_	196.3	0.4442	3.8620	H → L + 10	ICT	42
TYR295	S_11_	177.4	0.3142	2.0488	H-2 → L + 3	ICT	36
TRP286–TYR295	S_30_	179.9	0.5140	3.8191	H-5 → L+8	ECT	33
calcitriol-TRP286	S_2_	269.4	0.4954	3.6932	H → L + 1	ICT, ECT	84
calcitriol-TYR295	S_2_	268.0	0.4985	4.1706	H → L	ICT, ECT	46
calcitriol-TRP286-TYR295	S_3_	270.5	0.4545	2.2506	H → L + 2	ECT, ICT	61

a ICT: Intramolecular charge transfer; ECT: Intermolecular charge transfer.

b Normalized contribution percentage.

c H = HOMO; L = LUMO.

Intermolecular π-resonance within the calcitriol-TRP286 complex occurs at the interface between the molecules. This observation aligns with the NCI analysis, revealing a region of high electron density between TRP286’s indole ring and calcitriol (Figure 4).The HOMO orbital is delocalized over the polyene system of the ligand and the indole ring of TRP286, while the LUMO+2 orbital extends across both the conjugated polyene system of the ligand and the central region of TRP286. This distribution reflects a limited spatial separation between donor and acceptor orbitals, favoring intermolecular charge transfer. These features demonstrate that TYR295 functions neither as charge donor nor acceptor in the complex, contrasting with TRP286’s active role in the predominant electronic transition during ligand binding. TRP286 participates directly in electron transfer, with the resulting charge delocalization supporting intermolecular conjugation across the interface.

The predominant transition in the ternary complex (calcitriol with TRP286 and TYR295) corresponds to the S_0_ → S_3_ excitation (*n* = 1–45), exhibiting maximum absorption (λ_max_) at 270.5 nm (Table 4). This represents a bathochromic shift of 3.6 nm relative to isolated calcitriol (λ_max_ = 266.9 nm), accompanied by decreased oscillator strength (0.5465 → 0.4545). Individually, TRP286 and TYR295 residues show λ_max_ values of 196.3 and 177.4 nm, respectively. When complexed individually with calcitriol, their λ_max_ values become nearly identical, differing by only ∼1 nm (Table 4).

Notably, despite λ_max_ variations upon complexation (with individual residues or both), absorption occurs near isolated calcitriol’s wavelength. This highlights calcitriol’s dominant contribution to electronic transitions in complexed states, attributable to its conjugated polyene system acting as primary chromophore. A Natural Transition Orbital (NTO) analysis^57^ was performed to confirm the nature of the electronic excitations, which highlighted the greater electronic contribution of calcitriol transitions.

UV–vis spectra (Figure 7) were computed at the TD-ωB97*X*/6–311++G­(2df,p) level to analyze the calcitriol-aromatic residue complex at VDR’s active site. Spectra were generated with 20 nm full-width-at-half-maximum (fwhm) broadening and normalized. Absorption maxima correspond to spectral regions with higher densities of electronic excitations. Although individual transitions may exhibit low oscillator strengths, their cumulative effect produces more intense absorption bands than the strongest single transition. This is particularly evident in calcitriol-containing systems, where a shoulder adjacent to the main peak arises from a more intense excitation at longer wavelength (Figure 7).

**Figure 7:**
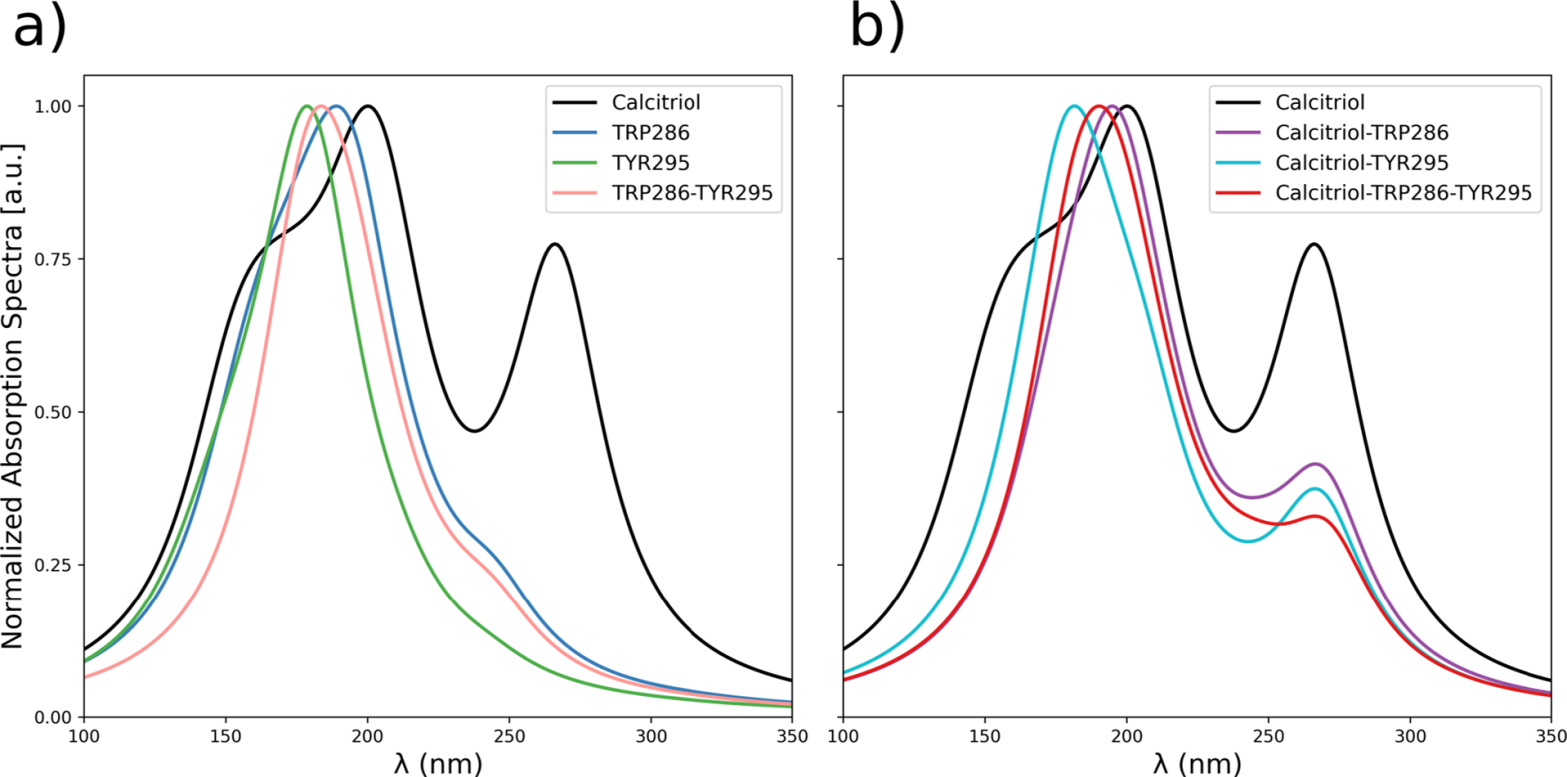
TD-ωB97*X*/6–311++G­(2df,p)-calculated UV–vis spectra of (a) bioactive calcitriol and isolated aromatic VDR residues (TRP286 and TYR295); (b) calcitriol-residue complex.

Figure 7A reveals a dominant, highly intense allowed π → π* transition for isolated TRP286 (blue spectrum). TYR295 (green) also exhibits a π → π* transition but with maximum absorption at a shorter wavelength. The complex of both aromatic residues (pink) shows an absorption maximum at an intermediate wavelength (184 nm) relative to the isolated residues, while maintaining high intensity.

Analysis of the UV–vis spectrum shows λ_max_ = 266 nm for the bioactive calcitriol conformation from system 25 (Figure 7B, black). Excited-state calculations indicate prominent π → π* transitions arising from unsaturated centers in calcitriol that form π orbitals.^58^ The conjugated system thus functions as the molecular chromophore, with absorption in the violet spectral region.

Complexation with TRP286 and TYR295 (red spectrum) reduces calcitriol’s main absorption peak intensity and induces a 10 nm hypsochromic shift (∼200 nm → 190 nm). Notably, the characteristic shoulder at 266 nm shows markedly decreased intensity upon complexation-whether with TRP286 (purple), TYR295 (light blue), or both residues (red). In the ternary complex (calcitriol-TRP286-TYR295), this shoulder’s absorption further diminishes, indicating a partially forbidden transition.

These spectral changes arise from the conjugated side chains of the aromatic residues. The intensity shift of the smaller peak corresponds to π → π* transitions from the aromatic systems (TRP286’s indole and TYR295’s phenolic rings), which constitute the predominant H → L+2 transition contributing 61% in the complex. This demonstrates TRP286’s significant influence on electronic transitions relative to isolated calcitriol (Figure 7).

Figure 8 displays total electron density mapped onto molecular electrostatic potential surfaces for calcitriol, TRP286, and TYR295 in the VDR-calcitriol complex (system 25 conformation). Electronic charge distributions were determined via Mulliken population analysis, quantifying each atom’s contribution to total charge in individual molecules and complexes.

**Figure 8:**
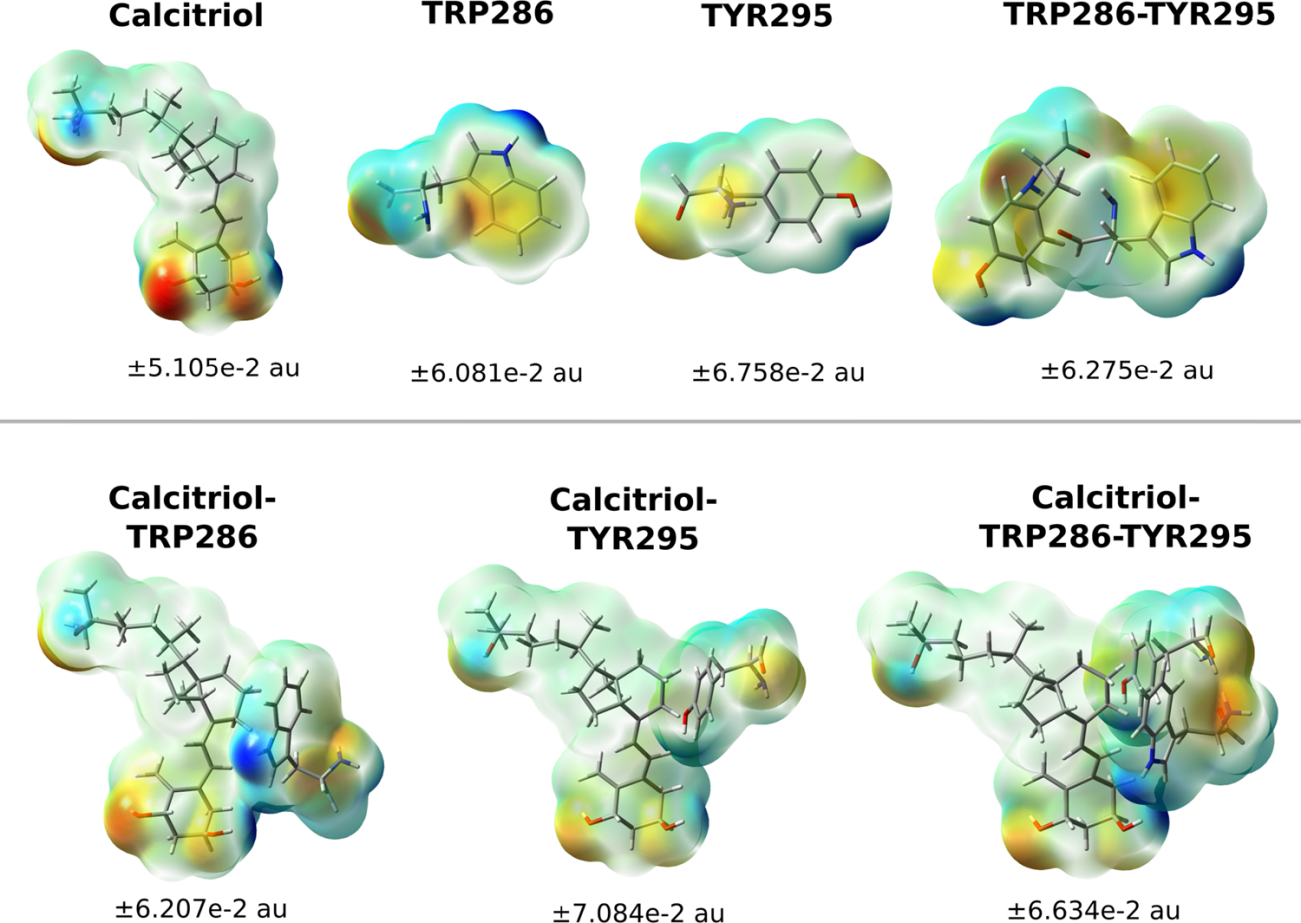
Molecular electrostatic potential (MEP) mapped onto electron density isosurfaces (0.001 au) for bioactive calcitriol and key aromatic VDR residues (TRP286, TYR295), computed at the ωB97*X*/6–311++G­(2df,p) level.

The isolated calcitriol molecule exhibits near-uniform electrostatic potential across most of its structure. Notable exceptions occur at hydroxyl oxygen atoms, which display electron-rich character (red-orange regions), and corresponding hydroxyl hydrogens, which appear deep blue indicating electron-deficient character.

The TRP286 residue shows yellowish coloration over its indole ring, indicating moderate electron enrichment from π-bonding. A red-orange region near the carbonyl group reflects high electron density at oxygen, contrasting with blue regions at nitrogen atoms indicating electron deficiency. TYR295 exhibits similar patterns but with an additional electron-rich region (comparable to oxygen sites) near its central nitrogen atom.

When complexed with both residues, calcitriol shows specific charge redistribution. The hydroxyl oxygens shift from red-orange to light orange, indicating reduced electron density. The yellowish π-electron density over TRP286’s indole ring becomes neutralized, demonstrating electron density reduction from complexation.

Conversely, the central region surrounding the conjugated triene, where amino acid side chains align with the ligand, displays light blue coloration suggesting balanced electrostatic potential. This results from donor–acceptor interactions enabling partial electron delocalization and charge sharing, potentially through π–π interactions or charge-transfer mechanisms. This distribution aligns with NCI analysis, revealing a pronounced hydrophobic channel encompassing the triene moiety that stabilizes the ligand within the VDR binding cavity.

## 4 Conclusions

This study successfully electronically determined the specific three-dimensional bioactive conformation of calcitriol within the vitamin D receptor active site cavity, thereby validating the methodological approach used. The results obtained for the protein–ligand complex closely align with experimental data, providing a deeper understanding of the intermolecular interactions and electronic transitions involved. Furthermore, the key interactions involved in the formation of the receptor–ligand complex were elucidated. Notably, the proximity of two aromatic amino acid residues, TRP286 and TYR295, to the electronic transition region of bioactive calcitriol induces alterations in the UV–vis spectrum. TD-DFT analysis indicates that calcitriol remains the dominant chromophore and that its main π → π* transition is slightly shifted upon interaction with TRP286 and TYR295. These effects, experimentally inaccessible due to the absence of UV–vis data for the holo complex, provide a detailed view of how these binding pocket residues influence at the excited-state behavior of calcitriol. Overall, this work fills a critical gap in the characterization of the VDR-calcitriol system by linking structure, interaction energetics, and excited-state properties. These findings offer valuable insights that can guide the rational design of novel calcitriol analogues with improved therapeutic profiles, especially in cases where experimental structural data on protein–ligand complexation are lacking.

## Data Availability

The protein structure used in this work was downloaded from the Protein Data Bank (PDB 1DB1) and is publicly available at https://www.rcsb.org/structure/1DB1. The data and scripts underlying this study are deposited and accessible at Repositório de Dados de Pesquisa da Unicamp (10.25824/redu/JZFFIL).
